# Water-Microdroplet-Driven Interface-Charged Chemistries

**DOI:** 10.1021/jacsau.4c00804

**Published:** 2024-11-01

**Authors:** Xiuquan Jia, Jianhan Wu, Feng Wang

**Affiliations:** † State Key Laboratory of Catalysis, Dalian Institute of Chemical Physics, Chinese Academy of Sciences, Dalian 116023, P. R. China; ‡ University of Chinese Academy of Sciences, Beijing 100049, P. R. China

**Keywords:** charged water microdroplets, contact electrification, electron transfer, ROS and H_2_ formation, N_2_ oxidation

## Abstract

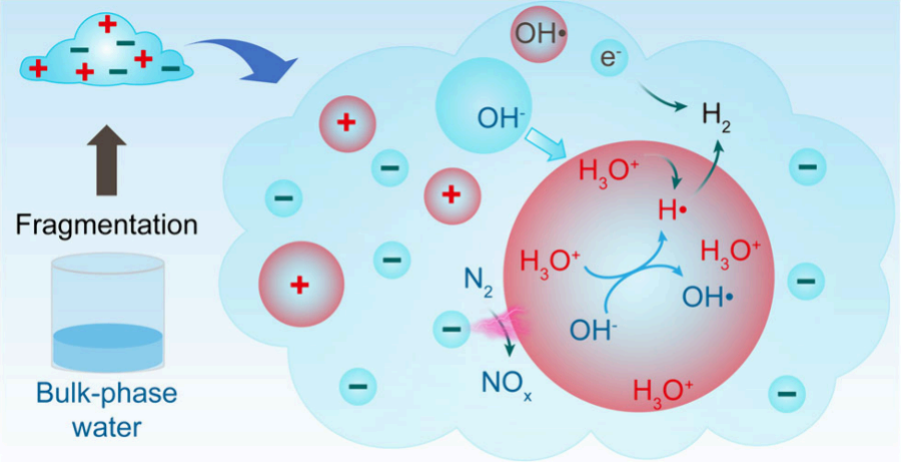

Water has made Earth a habitable planet by electrifying the troposphere. For example, the lightning caused by the electrification and discharge of cloudwater microdroplets is closely related to atmospheric chemistry. Recent work has revealed that a high electric field exists at the interface of water microdroplets, which is ∼3 orders of magnitude higher than the electric field that accounts for lightning. A surge of exotic redox reactions that were recently found over water microdroplets can be contributed by such an interfacial electric field. However, the role of net charge in microdroplet redox chemistry should not be ignored. In this Perspective, we show how redox reactions can be driven by electron transfer pathways in the electrification and discharge process of water microdroplets. Understanding and harnessing the origin and evolution of charged microdroplets are likely to lead to a paradigm shift of electrochemistry, which may play an overlooked role in geological and environmental chemistry.

## Introduction

Water microdroplets have shattered the paradigm of chemistry by driving a surge of chemical reactions that are otherwise sluggish or cannot take place under conventional bulk reaction conditions. Since the pioneering works by the Cooks group on the acceleration of bimolecular reactions in microdroplets^1^ and by the Mazumdar group on the charged microdroplet induced fragmentation reactions of analyte that is stable in bulk solution,^2^ this fascinating microdroplet chemistry field has stimulated strong interest. The ability to form high electric fields at the interface of water microdroplets^3−6^ combined with the inability to form a three-dimensional solvation shell around ions at the interface has been verified to lower the activation barrier^7−11^ and increase successful collision rates.^12,13^ Acceleration in many reactions is therefore feasible in microdroplets. Also, the high electric field can turn on redox reactions by enabling the spontaneous ionization of OH^–^ or other oxidizable solutes at water microdroplet interfaces.^14−21^ In contrast to the widely proposed kinetic factors, the thermodynamic origin of the microdroplet reactions remains elusive. The most prominent case might be that water and various organic solutes in microdroplets exhibit redox reactions that are thermodynamically unfavorable in bulk phase unless in the presence of energy input.^18,22−26^ Recent evidence has raised questions about the role of net charge in resolving this puzzle,^27,28^ with potentially widespread implications given the massive amount of charged water microdroplets both in nature and in the lab.

In 1892, the Nobel Laureate Philipp Lenard reported that when water breaks into tiny droplets, the negative and positive charges are separated,^29^ which is the earliest indication of water microdroplets formed with a net charge. In fact, net charge extensively exists for microdroplets prepared using ultrasonication,^30^ electrospray,^31^ splash,^32^ and gas nebulization.^33^ There are two main explanations for the origin of the net charge. Early work invoked ion transfer, but more recent works that verified the spontaneous redox reactions on water microdroplets indicate that electron transfer plays a prominent role. The pioneering works by Zare’s group presented the existence of spontaneous electron abstraction from the interfacial OH^–^ of sprayed water microdroplets, which accounted for the formation of H_2_O_2_.^25,34^ Zhang’s group was the first to demonstrate the strong spontaneous redox power of water microdroplets by investigating sprayed water microdroplets containing dissolved pyridine and viologen compounds,^15,18^ leading to a series of works that reflected the importance of microdroplets in electron-mediated atmospheric redox chemistry^17,35^ and radical-based organic synthesis.^16,19,21,36,37^ Wang et al. recently investigated the size-dependent charge separation between two separating water microdroplets produced by ultrasonic atomization, in which they described a propensity for interfacial electrons to move from large microdroplets to small microdroplets.^38^ Wang’s group also found that electron transfer predominates on hydrophobic solid surfaces, leading to the ionization of water in contact with the hydrophobic surface.^39^ Liang’s group and Zare verified the electron transfer pathway in the contact electrification process at water–silica interfaces by observing the formation of H_2_O_2_ on microdroplets as well as a concomitant current flow.^40^ Electron transfer pathways also contribute to the formation of charged microdroplets in water–oil emulsions^41^ via charge transfer across C–H···O hydrogen bonds at water–oil interfaces.^42^ Recent works by us and Zare’s group^28,43^ and a more recent work by Cao’s group^44^ showed that water–oil emulsions enable the observation of water-derived H_2_ formation as the chemical fate of the electrons pulled from the water microdroplet interface. Apart from the various electrification processes that generate net charge, Head-Gordon’s group^27^ provided theoretical evidence that subsequent mutual neutralization reactions on or between charged water microdroplets, as proposed by Colussi,^45^ can result in electron transfer pathways as well. Very recent works by Zare’s group^33^ and Banerjee’s group^46^ showed that electron transfer can even exist between the surrounding air molecules due to their ionization induced by the charged water microdroplets. Given the fundamental role of electron transfer in chemistry,^47^ the formation of charged microdroplets and their evolution should be related to the dramatic alteration of the reaction thermodynamics compared to the bulk phase.

Zhang’s group covered a lot of ground in the previous Perspective on microdroplet chemistry.^19^ Since then, considerable progress in microdroplet chemistry has been made. One of the important advancements is understanding the role of net charge in the interfacial chemical reactions over water microdroplets. The goal of this Perspective is to highlight specific examples of charged microdroplet chemistry. By using selected examples and drawing common themes between different microdroplet reactions, we aim to show how these commonalities can be used to understand and harness the redox chemical impacts of net charge in water microdroplets.

The extensive interest in the redox chemistry of water microdroplets originated from the observation of reactive oxygen species (ROS) such as ·OH and H_2_O_2_ formed in microdroplets. However, the origin of the ROS has been a controversial topic, with clear evidence that H_2_O_2_ can arise from external gases in contact with the microdroplet surface. It is essential to consider the exogenous environmental factors that might contribute to the redox reactions over microdroplets. Mishra and co-workers revisited the formation of H_2_O_2_ at the water microdroplet interface generated by sonic spray,^48^ and they found that ozone can be the major contributor to the formation of H_2_O_2_ in sprayed water microdroplets. Another study by Eatoo and Mishra demonstrated that the partial reduction of dissolved oxygen by the oxidizable solid surface could also lead to the conspicuous formation of H_2_O_2_.^49^ Based on their careful experimental analyses, the presence of ozone or ambient oxygen should indeed be capable of contributing to or even dominating H_2_O_2_ formation over the microdroplets. Nevertheless, ^18^O labeling experiments verified that the water molecule at the droplet interface is the oxygen source of the H_2_O_2_ in microdroplets, which does not necessarily require ozone.^40,50^ A more recent topic under debate came from the direct detection of ·OH by mass spectrometry, in which a peak was observed at *m*/*z* 36 and initially attributed to (H_2_O)_2_^+•^^22^ or ·OH–H_3_O^+^.^23^ Chen and Williams recently investigated the aqueous droplets formed both by nanoelectrospray and a vibrating mesh nebulizer but did not observe this peak after obviating interference from ammonium.^51^ The absence of these species might be ascribed to the absence of nebulizing gas or to the different mass spectrometric sensitivity or skimmer potential that might break down such complexes. Additional differences in various studies could be reconciled by taking into account the various bath gases, the relative humidity, the pH, the dissolved salts, the detection limits, and the specific role of electron scavengers.^20,52,53^ Therefore, in order to verify ROS formation in microdroplets, supplementary measurements with results that agree with one another should provide convincing results.

## Contact Electrification at Water Microdroplet Interfaces

Contact electrification occurs at almost any material contact interface. The work of Chen et al. confirmed the formation of ROS in the initial stage of the interface formation between water microdroplets and silica.^40^ Notably, the generation of ROS is synchronized with a current flow that is sufficient to account for the electron transfer required for the generation of ROS by the ionization of OH^–^. ^18^O labeling experiments verified that both water molecules and the hydroxyl groups on the SiO_2_ substrate are the origin of the oxygen atoms for the generation of H_2_O_2_.

**Figure 1:**
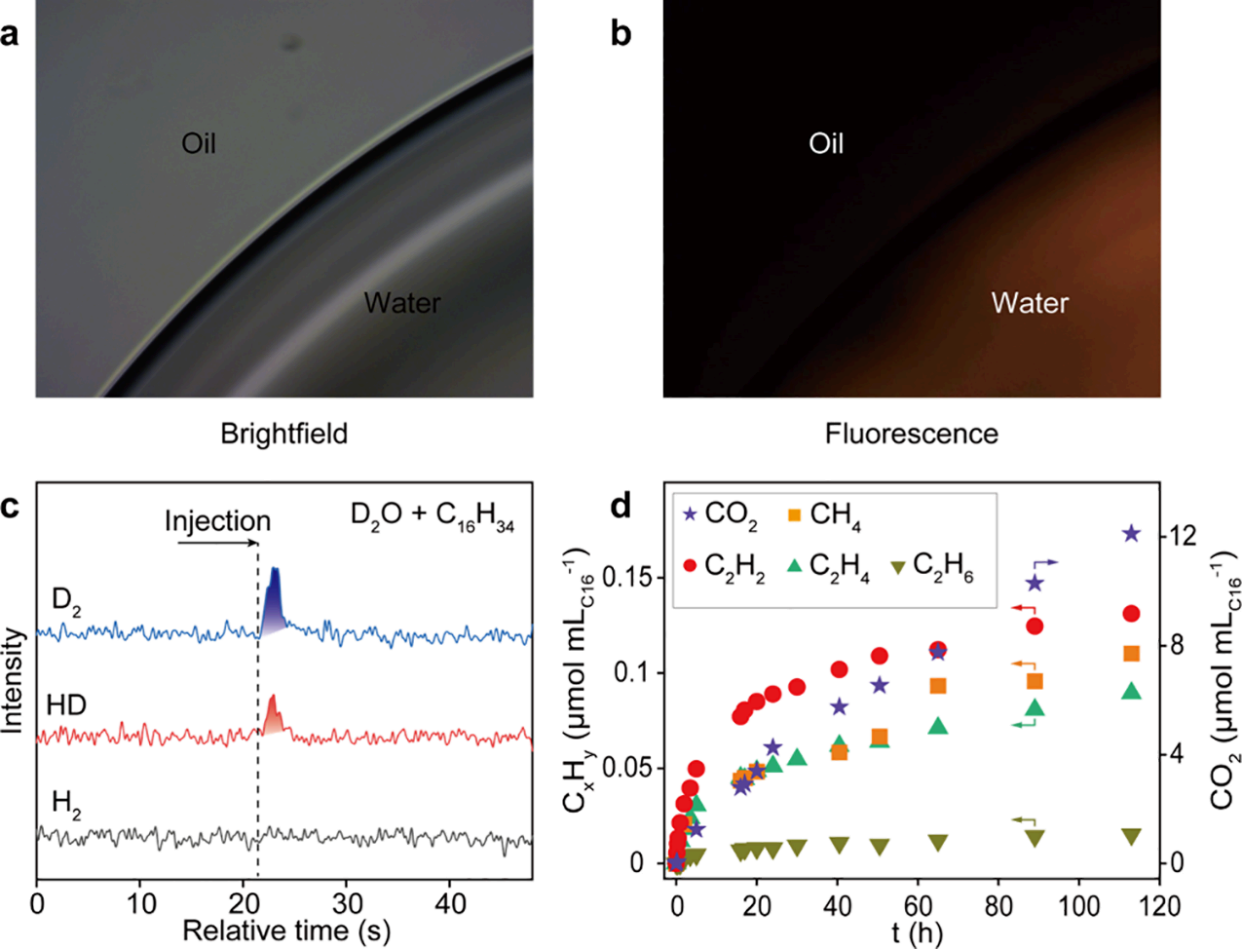
Evolution of ROS, H_2_, and gaseous C_1_–C_2_ products. (a, b) Fluorescence microscopy images of ROS generation in a hexadecane-dispersed water droplet. (c) Mass spectrometric detection of hydrogen gas in the gas phase above evacuated D_2_O/hexadecane emulsion. (d) Time course of C_1_–C_2_ products from the evacuated emulsion. Adapted from ref (43). Copyright 2023 American Chemical Society.

While the generation of ROS can be explained well by the contact electrification at water–solid interfaces, the question remained unsettled regarding the chemical fate of the electrons that are separated from the ROS. Unraveling this question is a prerequisite for understanding the thermodynamics of redox reactions. There have been studies showing that the electrons are taken by some reducible species in air, and there are also studies guessing that H_2_ might be formed by reduction of water. Our group and the Zare group recently codiscovered that aqueous microdroplets in emulsified water/hexadecane (C_16_H_34_) mixtures can not only form ROS (Figure 1a,b) but also generate H_2_.^43^ In this work, we extended the contact-electric redox chemistry to highly deformable water–oil microdroplet interfaces by describing the spontaneous generation of H_2_ and ROS from microdroplets of water in contact with the model oil hydrocarbon hexadecane. D_2_ and DH instead of H_2_ were observed upon replacing H_2_O with D_2_O (Figure 1c), confirming that H_2_ primarily comes from water but not the hydrocarbon. The H_2_ evolution reaction proceeds only in the fresh emulsion. The subsequent generation of H_2_ might be hindered by the accumulation of aqueous ROS and other oxidative products as competitive electron acceptors or H_2_ scavengers at the water interfaces.

The microdroplets of water in contact with the hexadecane also led to the evolution of CO_2_ and the production of short-chain hydrocarbons (mainly C_1_ and C_2_; Figure 1d). Such gaseous hydrocarbons as important energy resources and greenhouse gases are extensively generated from abandoned oil wells. However, their light hydrocarbon concentration ratios (C_2–4_/C_1_) are much higher than those from microbial sources, pointing to an abiotic origin.^54^ We suggest that water–oil microdroplet chemistry may be important for interpreting and manipulating these oil transformations. More recently, Cao’s group investigated the thermal geochemical systems where *n*-C_20_H_42_, H_2_O, and K-feldspar interact.^44^ It was observed that water microdroplets spontaneously formed at 150–165 °C in oil phases also led to the generation of ·OH and CO_2_ as well as enhanced formation of hydrocarbon gases and H_2_. Besides, upon replacing H_2_O with D_2_O, the observed incorporation of the deuterium of D_2_O into the newly formed gases, liquid hydrocarbons, and *n*-C_20_H_42_ substrate indicated the presence of water-derived hydrogen radical at the microdroplet water–oil interfaces. Hence, the generation of ·H and H_2_ from water microdroplets in emulsified water–oil mixtures not only is an essential reaction step in the microdroplet redox chemistry but also has significant implications for understanding the chemistry of the geosphere.

**Figure 2:**
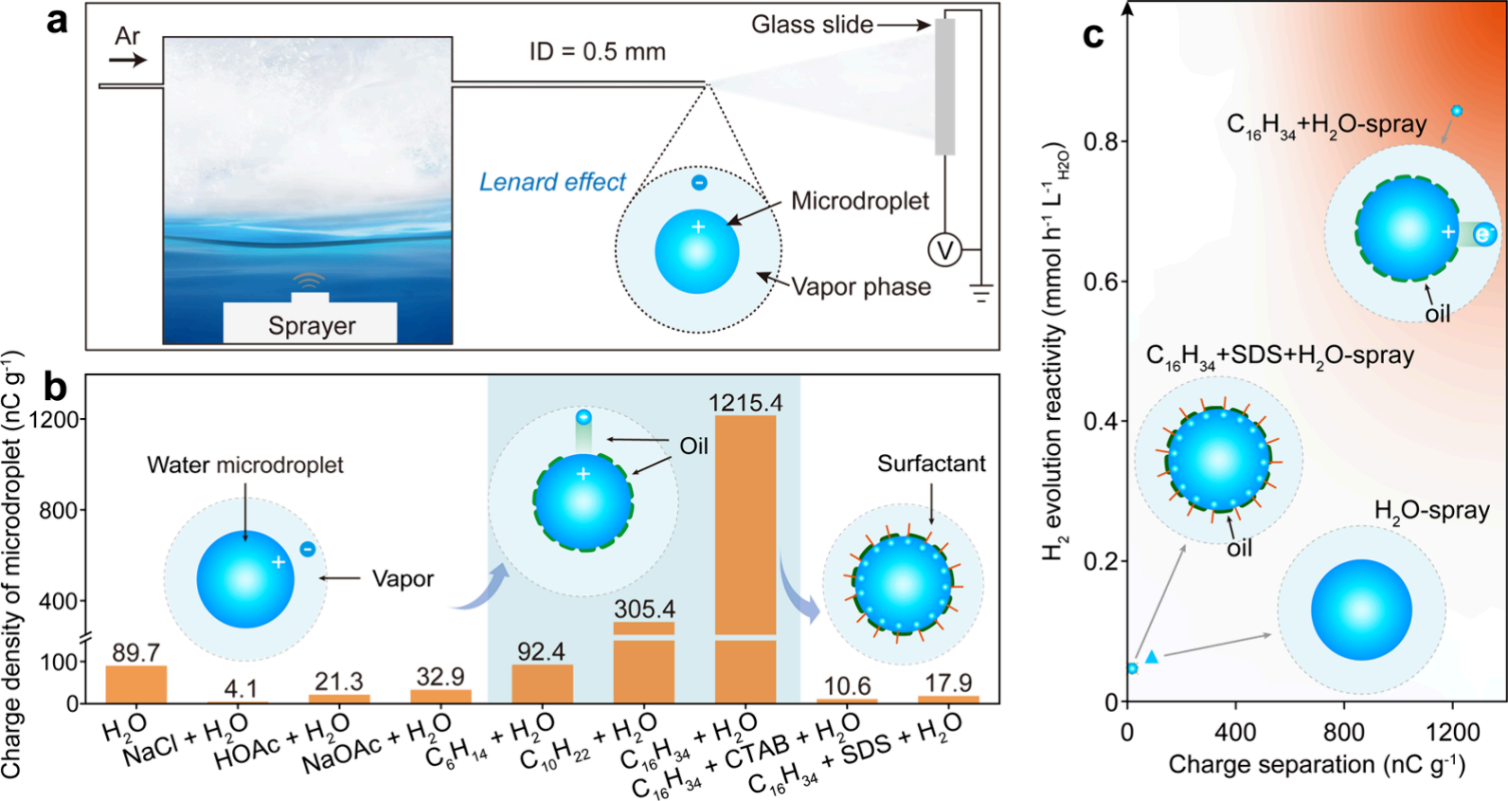
Charge and H_2_ formation of sprayed hexadecane/water microdroplets. (a) Experimental setup for charge measurements. (b) Measured electric charge density of microdroplets with additives. (c) Relationship between charge separation of microdroplets and spray-sourced H_2_ evolution reactivity. Adapted from ref (28). Copyright 2024 American Chemical Society.

Our recent work has shown that this redox reaction of water molecules can be further enhanced by contact electrification with hydrophobic surfaces.^28^ We prepared oil–water microdroplets by ultrasonically atomizing a mixture of 0.5 mL of hexadecane and 70 mL of water and investigated the spray electrification of microdroplets by measuring the accumulated charge of microdroplets collected (Figure 2a). Our results showed that the microdroplets tended to carry positive charges, while the vapor phase tended to be negatively charged. Besides, we observed that when an oil–water mixture is sprayed, the contact electrification between oil and water can be employed to extract a negative charge from the sprayed microdroplets (Figure 2b). This results in an ∼13-fold increase of net charge in comparison with an ultrapure water spray (Figure 2b). The enhanced net charge gives a factor of ∼16 improvement in the spray-sourced hydrogen evolution from water (Figure 2c). This indicates enhanced electron abstraction from sprayed water microdroplets by contact electrification between oil and water. The addition of surfactants such as hexadecyltrimethylammonium bromide (CTAB), sodium dodecyl sulfonate (SDS), and nonionic surfactants to occupy the interfacial region instead of oil led to a significant reduction in the charge of microdroplets (Figure 2b). This reduction in the charge was accompanied by a decrease in the H_2_ evolution reactivity (Figure 2c), indicating inhibition of electron abstraction from water microdroplets. Inhibition of the electron abstraction via the addition of other ionic additives like sodium chloride also inhibited the evolution of H_2_, which indicates a low reactivity of H_2_ formation for seawater microdroplets. Intriguingly, we observed that replenishing electrons to seawater microdroplets via contact electrification with pyrogenic carbon can restore the activity of H_2_ formation by the galvanic coupling between the microdroplet water–carbon interfaces and the microdroplet water–vapor interfaces.^55^ The above works emphasize the chemical effect of electron transfer in the initial stage of charged microdroplet formation.

## Mutual Neutralization of Interfacial Hydronium and Hydroxide

**Figure 3:**
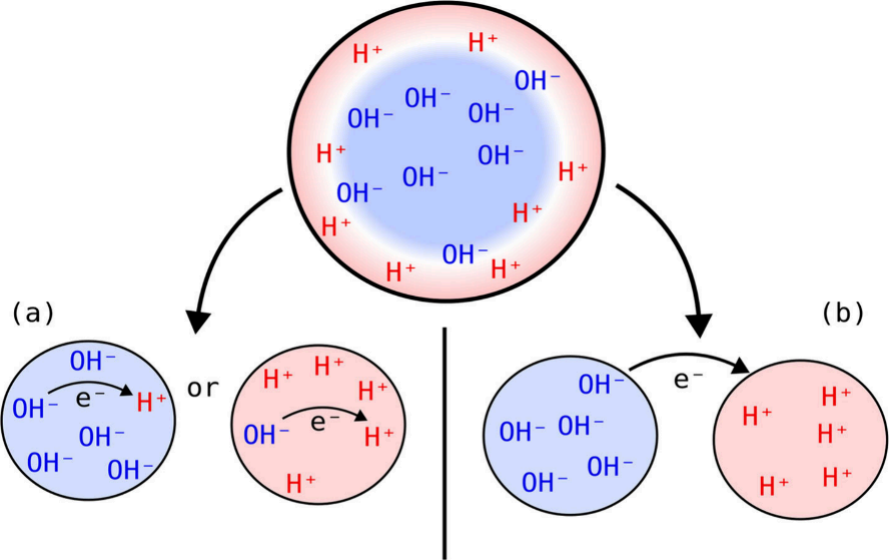
Schematic illustration of two mechanisms for redox chemistry in charged microdroplets. Adapted from ref (27). CC BY 4.0.

Apart from the electron transfer during the formation of charged microdroplets, we consider an alternative electron transfer pathway initiated by the enhanced autoionization of interfacial water dimer [(H_2_O)_2_]_i_:^56^
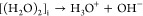
1Mutual neutralization of H_3_O^+^ and OH^–^ ions is a fundamental chemical reaction. However, a rich variety of electron transfer pathways can exist behind the seemingly simple textbook process of neutral water molecule generation. The OH^–^ and H_3_O^+^ cluster together at the interface, with a preference for one or the other to be at the extreme outside. This charge separation provides a strong localized electric field that drives the loss of electrons from OH^–^^25^ to form ·OH and from (H_2_O)_2_^57^ to form ·OH–H_3_O^+^. This may also account for the formation of e_aq_^–^, which can be accepted by H^+^ ions to form ·H. We also have the mutual neutralization process H^+^ + OH^–^ → ·H + ·OH occurring at the interface, as proposed by Colussi.^45^ Hence, at the interface but not the bulk or the interior of the droplet, we have
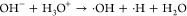
2This reaction is aided by the partial desolvation of H^+^ and OH^–^ at the interface of microdroplets.^14^ The variation of redox potentials at the air–water interface relative to the bulk solution is consistent with the theoretical study by Martins-Costa and Ruiz-Lopez in 2023.^58^ They revealed that local electric field at interface, which is not larger than that in bulk solution, cannot explain the observed microdroplet chemistry. The electrostatic potential was also shown to be a key factor,^58^ implying the potential role of net charge of water microdroplets. Head-Gordon’s group recently provided theoretical evidence for the potential of electron transfer as a result of net charge solely derived from additional OH^–^ or H^+^ ions (Figure 3).^27^ The mutual neutralization process H^+^ + OH^–^ → ·H + ·OH is strongly disfavored by the positive enthalpy (Δ*H* = 107 kcal/mol) under neutral bulk conditions. However, in a droplet, the net charge contributed by additional OH^–^ or H^+^ ions alters the hydration enthalpy of OH^–^ and H^+^ ions. They found that at ∼20% to 50% of the Rayleigh limit of droplet charge, the hydration enthalpy of both OH^–^ and H^+^ decreases by >50 kcal/mol, such that electron transfer from OH^–^ to H^+^ becomes thermodynamically favorable. This work illustrates an interesting point that the Coulomb repulsion contributed by the net charge can make the electrons of OH^–^ more weakly bound and attracted to H^+^ more strongly. A recent work by Bogot et al. provided experimental evidence for the electron transfer mechanism of mutual neutralization by realizing the interaction in merged beams of two ionic species with near-zero relative velocity.^59^ They identified predominant H_2_O + ·OH + ·H and 2(·OH) + H_2_ product channels and attributed them to an electron transfer mechanism. Additional experiments should be required to investigate the relevance of these electron transfer reaction pathways of gas-phase ions to mutual neutralization in the microdroplets.

## Discharge around Charged Microdroplets

Discharge of water microdroplets in clouds is known to strongly impact the chemistry on Earth by causing lightning,^60,61^ where temperatures near 30,000 K and pressures on the order of 8 atm have been observed.^62^ There has been a huge challenge to understand the fascinating chemistry that goes with this capricious phenomenon of cloudwater.^63^ For example, atmospheric N_2_ fixation has been recognized as a typical high-temperature chemistry, in which the role of water should be important but is not clear.^64^ Very recent works advanced our knowledge in this field by showing that charged water microdroplets can lead to the transformation of N_2_ via the ionization of the surrounding air at ambient temperature. Recently, Banerjee and co-workers found that water microdroplets generated by sonic spray, humidifier, spray bottle, and steamer can spontaneously generate nitrogen oxide under ambient conditions.^46^ This was the first study to reveal the electrical breakdown of air (microlightning) at the charged water microdroplet interface in the absence of an applied potential. In this study, they proposed that the strong intrinsic electric field at the water microdroplet surface could lead to electrical breakdown of air at the air–water interface, resulting in electron-rich microdroplets and electron-deficient air. They suggested that the intrinsic corona discharge at the air–water interface likely activates the N≡N bonds in molecular nitrogen, forming nitrogen oxides and acids. In the work by Zare’s group,^33^ gas-assisted water atomization was found to produce negatively charged droplets, which can further undergo water–water electrification upon fission to form larger positively charged microdroplets and smaller negatively charged microdroplets. The intensity of the local electric field formed at that moment of droplet separation can reach approximately 3 × 10^9^ V/m. In addition, the observation of N_2_^+^, NO^+^, O_2_^+^, and NO_2_^+^ formed by the ionization of surrounding air indicates an occurrence of microlightning. Thus, observational agreement on the ability of charged water microdroplets to drive N_2_ oxidation can be inferred, which can be indirect proof of the existence of a high electric field at the microdroplet interface or during the droplet disintegration process. However, Banerjee and co-workers also mentioned that the amount of NO_*x*_ produced by the spray process might be limited to the ppt or sub-ppb level, which is much below the safe level of NO_*x*_ or below the NO_*x*_ level in unpolluted air. Such low abundance is inadequate for the consideration of this water-microdroplet-sourced reactive nitrogen as a fundamental factor in evaluating the reactive nitrogen availability in ecosystems.

## Outlook

The net charge of water microdroplets is not only a result of interfacial electron transfer by contact electrification but also a driving force for the electron transfer reactions of ions and neutral molecules on or around the microdroplet surfaces. Once we have the ability to precisely describe the origin and evolution of charged water microdroplets, their implications for the biological, environmental, and geological science can be predicted with less uncertainty. However, it is difficult to investigate one electron transfer pathway without interference from the others. For example, contact electrification can occur at both the water–solid and other interfaces. Also, ROS formation by contact electrification at water–solid interfaces can be investigated with a fluorescence microscopy setup for imaging a single water microdroplet confined in the microfluidic or microwell chip.^40,65^ By contrast, the experimental setup for investigating the effect of water–gas and water–water electrification could hardly avoid the interference from the water–solid interfaces. In order to disentangle the contribution of water–solid electrification from that of water–gas or water–water electrification, updated techniques for the levitation and atomization of liquid water are required. Furthermore, the electron transfer in the electrification process should be controlled in order to avoid interference in the experimental investigations on subsequent discharge or mutual neutralization of charged microdroplets. From the perspective of disentangling the contribution of electrification from that of discharge, the method for charged microdroplet preparation in the work of Williams and co-workers should be valuable because there is little or no ROS formation in the electrification process.^51^ Methods for distinguishing or controlling the multiple electron transfer pathways in experimental settings remain to be developed, which would strengthen the practical understanding of microdroplet chemistry and its applications.

One key issue in microdroplet chemistry is the reaction scale, which hitherto has been too small to play a predominant role in most cases. This is partially caused by an inefficient buildup of chemical potential from the energy that accounts for the formation of microdroplets. Given the feasibility to control the electrification and discharge as well as the kinematics and kinetics of charged water microdroplets, the thermodynamics of microdroplet chemistry is likely to be modulated by natural and artificial factors. Therefore, scaling up the formation of products in certain scenarios would be possible to markedly impact the environmental and geological processes. In the case of improved energy utilization efficiency, charged microdroplet chemistry might offer an electrochemical strategy to address challenging problems of environmental and industrial importance like CO_2_ emissions reduction and sequestration, waste upcycling, and clean water generation. In this regard, the defining features of a charged water microdroplet that distinguish it from other charge carriers can be the foundation of unexpected paradigms of electrochemistry.
